# Phytochemical Combination PB125 Activates the Nrf2 Pathway and Induces Cellular Protection against Oxidative Injury

**DOI:** 10.3390/antiox8050119

**Published:** 2019-05-03

**Authors:** Brooks M. Hybertson, Bifeng Gao, Swapan Bose, Joe M. McCord

**Affiliations:** 1 Pathways Bioscience, Aurora, CO 80045, USA; bifeng.gao@ucdenver.edu (B.G.); swapanbose91@gmail.com (S.B.); joe.mccord@ucdenver.edu (J.M.M.); 2 Department of Medicine, Division of Pulmonary Sciences and Critical Care Medicine, University of Colorado Anschutz Medical Campus, Aurora, CO 80045, USA

**Keywords:** Nrf2, KEAP1, oxidative stress, aging, C9orf72, PCSK9, APOL1, BHMT, CBS, CYP1A1, MAT1A, NOS3, VGF

## Abstract

Bioactive phytochemicals in *Rosmarinus officinalis*, *Withania somnifera*, and *Sophora japonica* have a long history of human use to promote health. In this study we examined the cellular effects of a combination of extracts from these plant sources based on specified levels of their carnosol/carnosic acid, withaferin A, and luteolin levels, respectively. Individually, these bioactive compounds have previously been shown to activate the nuclear factor erythroid 2-related factor 2 (Nrf2) transcription factor, which binds to the antioxidant response element (ARE) and regulates the expression of a wide variety of cytoprotective genes. We found that combinations of these three plant extracts act synergistically to activate the Nrf2 pathway, and we identified an optimized combination of the three agents which we named PB125 for use as a dietary supplement. Using microarray, quantitative reverse transcription-PCR, and RNA-seq technologies, we examined the gene expression induced by PB125 in HepG2 (hepatocellular carcinoma) cells, including canonical Nrf2-regulated genes, noncanonical Nrf2-regulated genes, and genes which appear to be regulated by non-Nrf2 mechanisms. Ingenuity Pathway Analysis identified Nrf2 as the primary pathway for gene expression changes by PB125. Pretreatment with PB125 protected cultured HepG2 cells against an oxidative stress challenge caused by cumene hydroperoxide exposure, by both cell viability and cell injury measurements. In summary, PB125 is a phytochemical dietary supplement comprised of extracts of three ingredients, *Rosmarinus officinalis*, *Withania somnifera*, and *Sophora japonica*, with specified levels of carnosol/carnosic acid, withaferin A, and luteolin, respectively. Each ingredient contributes to the activation of the Nrf2 pathway in unique ways, which leads to upregulation of cytoprotective genes and protection of cells against oxidative stress and supports the use of PB125 as a dietary supplement to promote healthy aging.

## 1. Introduction

Oxidative stress and the diminishment of the body’s antioxidant defenses are usually associated with aging and with a variety of disorders and diseases [1,2,3,4,5]. Dietary composition has significant influence on the body’s ability to fight against oxidative stress, which is one of the ways that dietary intake plays a role in healthy aging [6,7]. In the past this was largely attributed to direct scavenging of oxidants by compounds consumed in the diet [8,9,10], but in recent years attribution has shifted to endogenous protection mechanisms and to understanding the health benefits of dietary components based on their ability to activate endogenous defenses, for example by inducing the increased expression of antioxidant enzyme genes [11,12,13,14].

Nuclear factor erythroid 2-related factor 2 (Nrf2) is a transcription factor that regulates the gene expression of a wide variety of cytoprotective phase II detoxification enzymes and antioxidant enzymes through its binding to the antioxidant-responsive element (ARE) in the promoter region of these genes. Relevant to oxidative stress, the ARE is a promoter element found to regulate expression of many antioxidant enzymes [15,16,17,18,19,20,21,22,23]. Here we describe a novel three-component Nrf2 activator, PB125, and examine its effects on gene expression using GeneChip, RNA-seq and quantitative PCR technologies, comparing the results with three other Nrf2 activators. For the canonical Nrf2 dependent gene *HMOX1* we also measured heme oxygenase-1 protein levels (HMOX1) after PB125 treatment. We also demonstrate protection of HepG2 by PB125 against an oxidative stress challenge caused by cumene hydroperoxide exposure.

The functionality of Nrf2 cytoprotective signaling appears to diminish with age [24,25,26,27]. This leads to the unfortunate situation where aging-related stressors occur during a time of diminished protective responses [27,28]. Studies in both rats and people indicated significantly lower nuclear Nrf2 levels in old subjects compared to young subjects [24,27], and decreased capabilities for antioxidant defenses and for repair mechanisms [27].

In the present work, we sought to develop a dietary supplement named PB125^®^ to activate the Nrf2 pathway to promote healthy aging. PB125 is based on a combination of compounds found in *Rosmarinus officinalis*, *Withania somnifera*, and *Sophora japonica* (particularly carnosol/carnosic acid, withaferin A, and luteolin) [29,30,31,32,33,34,35,36,37,38,39,40,41,42,43,44,45,46,47]. The rationale for selection of these agents was based on their individual effects on the Nrf2 activation pathway and their synergistic effects when used in combination. Since the objective was to formulate for dietary usage, additional selection criteria included natural plant sources, established safety for human consumption, and prior evidence of health benefits from the individual dietary agents [29,34,42,48,49,50,51,52,53,54].

Rosemary has been used to treat a variety of disorders [1], with emphasis on anti-inflammatory [55], antioxidant [45,46,47], and antimicrobial benefits [56,57]. Ashwagandha has been utilized for immunomodulatory [58], anti-tumor [59], neurological [60], anti-inflammatory [53], antioxidant [37], and other benefits [61]. Luteolin is a bioflavanoid flavone compound commonly consumed in the human diet from multiple food sources [50,62,63,64,65] and is frequently utilized as a dietary supplement with emphasis on its antioxidant [43], neurological [41], and anti-inflammatory benefits [40,49,62]. Each of the three plant extracts in PB125 is standardized to contain active compounds (carnosic acid/carnosol, withaferin A, and luteolin) that are known Nrf2 activators, are amenable to analysis, and enable reproducible preparation of the combination.

The rosemary extract contains carnosic acid, which may be metabolically interconverted with carnosol, and was chosen for several unusual properties. Carnosic acid easily crosses the blood-brain barrier to upregulate endogenous antioxidant enzymes via activation of the Nrf2 transcriptional pathway and has been shown both in vitro and in vivo in mouse models to reduce brain injury occurring after sublethal cyanide poisoning [66] or after middle cerebral artery occlusion and reperfusion, significantly decreasing infarct volume by 33% [32]. A problem pointed out by Satoh and Lipton [67,68] is that systemically administered electrophiles can react with thiols such as glutathione before reaching their intended targets in the brain. They reasoned that a compound that functions as a pro-drug and converts to an electrophile by oxidation after reaching the intended target might be more desirable. Carnosic acid is such a compound, oxidizing spontaneously under conditions of oxidative stress to the more electrophilic quinone [32] that rearranges to form carnosol, thus having an advantage over direct antioxidant molecules because its action is more sustained and amplified by its conversion to a Nrf2 activator after reaching its target [66]. Thus, carnosol activates the Nrf2 pathway as an electrophilic compound reacting more specifically with cysteine-151 of Kelch-like ECH-associated protein 1 (Keap1, inhibitor of Nrf2) and releasing Nrf2, the first step in its journey to the nucleus, potentially avoiding the side effects associated with less discriminate electrophilic Nrf2 activators such as dimethylfumarate and curcumin [68,69].

*Withania somnifera*, better known as ashwagandha, contains a group of compounds known as including the increasingly studied compound withaferin A. The safety of ashwagandha extracts has been established in pre-clinical models and has been reviewed recently in the context of its possible activity in cancer chemoprevention and therapy [70]. Withaferin A activates Nrf2 in a Keap1-independent manner via the PTEN/PI3K/Akt pathway, unlike the prototypical Nrf2 inducers sulforaphane and CDDO-Im [71], and importantly, different from carnosic acid. Metformin, which is first-line treatment for type II diabetes and metabolic syndrome, and rapamycin, used to prevent transplant rejection, are two FDA-approved drugs that exhibit significant anti-cancer and anti-aging properties beyond their current clinical applications. Both drugs inhibit the mTOR pathway, which is involved in senescence and longevity [72]. However, each faces issues with approval for off-label, prophylactic use due to adverse effects. Aliper et al. [73] recently applied bioinformatic approaches to map the gene- and pathway-level signatures of metformin and rapamycin and screened for matches among over 800 natural compounds. They also analyzed the safety of each compound with an ensemble of deep neural network classifiers. The analyses revealed many compounds similar to either metformin or rapamycin, but only withaferin A and one other compound had strong similarities to both. Withaferin A was the top-scoring compound for gene expression similarity to metformin using the statistical approach and also displayed significant pathway- and gene-level similarity to rapamycin using both the pathway activation approach and the deep learning approach. We believe these similarities of the transcriptome effects of withaferin A to metformin and rapamycin (but without the adverse side effects) go beyond Nrf2 activation, and bring desirable properties to PB125.

Luteolin is a relatively good activator of Nrf2 by a PI3K-dependent mechanism [40], but was chosen as our third ingredient primarily for its ability to favorably affect Nrf2 activity by additional mechanisms completely different from those discussed above. Luteolin is a bioflavanoid flavone compound commonly consumed in the human diet from multiple food sources with reported antioxidant benefits [43]. Zuo et al. [74] found that luteolin could nearly double Nrf2 mRNA production, not by gene induction but by relieving epigenetic silencing of the Nrf2 promoter through reducing CpG methylation of the Nrf2 promoter region. Yet another action of luteolin that indirectly supports Nrf2 activity is the fact that luteolin potently inhibits GSK3B [75], the enzyme responsible for activation of the Fyn kinase that phosphorylates nuclear Nrf2 at tyrosine 586 [76] causing Nrf2 to be ejected from the nucleus, effectively shutting down Nrf2-dependent transcription. By preventing the activation of Fyn, the limited supply of Nrf2 available in aging cells may remain in the nucleus for a longer time, compensating in part for the short supply. Luteolin is also a potent inhibitor of xanthine oxidase [77], a superoxide radical-producing enzyme known to participate in diseases such as gout and reperfusion injury [78]. Another unusual and potentially useful property of luteolin has recently been reported by Son et al. [79]. They found luteolin can “normally” activate Nrf2 transcription in a human bronchial epithelial-derived cell line, BEAS-2B, protecting the cells from malignant transformation by hexadentate chromium, Cr(VI). If the cells are first transformed by Cr(VI), however, their Nrf2 becomes constitutively expressed, as is seen in several types of cancers. When the Cr(VI)-transformed cells were treated with luteolin at the same concentration, their Nrf2 expression was dramatically inhibited and HMOX1 expression declined [79]. Chian et al. [80] found that oral administration of luteolin, either alone or combined with intraperitoneal injection of the cytotoxic drug cisplatin, greatly inhibited the growth of xenograft tumors from non-small-cell lung cancer (NSCLC) cell line A549 cells grown subcutaneously in athymic nude mice. Thus luteolin appears to be able to activate Nrf2 in non-cancerous tissues while deactivating it in those cancers that constitutively express Nrf2, supporting its selection for use as a Nrf2-related dietary ingredient.

In short, in the current work we studied three well known dietary ingredients with the objective of identifying a combination (which we named PB125) that synergistically activates the Nrf2 cell signaling pathway. Then, because the PB125 combination of ingredients was more potent than the individual ingredients by themselves, we studied the ability of the PB125 combination to upregulate antioxidant, anti-inflammatory, and other cell protective genes, and protect cultured cells against oxidative stress.

## 2. Materials and Methods

### 2.1. Materials and Reagents

Plant extracts: rosemary extract from *Rosmarinus officinalis* (standardized to 6% carnosol; 15% carnosic acid) was obtained from Flavex (Rehlingen, Germany), ashwagandha extract from *Withania somnifera* (standardized to 2% withaferin A) was obtained from Verdure Sciences (Noblesville, IN, USA), ginger extract from *Zingiber officinalis* (standardized to 20% gingerols) was obtained from Flavex (Rehlingen, Germany), and luteolin (standardized to 98% luteolin, from *Sophora japonica*) was obtained from Jiaherb (Pine Brook, NJ, USA). For making PB125 solutions, the rosemary, ashwagandha, and luteolin powders were mixed at a 15:5:2 ratio by mass, then extracted at 50 mg of mixed powder per mL in ethanol overnight and the supernatant isolated. For gene expression comparisons a similar composition, PB123, containing rosemary, ginger, and luteolin was prepared at a 10:5:1 ratio [81]. Dibenzoylmethane (DBM) was obtained from Sigma (Saint Louis, MO, USA). Protandim^®^ (LifeVantage, Sandy, UT, USA) was extracted and analyzed as previously described [82]. Cell culture: media and antibiotics were purchased from Thermo Fisher Scientific (Waltham, MA, USA).

### 2.2. Cell Culture

We utilized the human HepG2 cell line (hepatocellular carcinoma) for genomic and cytoprotection assays and we utilized a HepG2 cell line that had been stably transfected with a Nrf2-responsive, ARE-regulated firefly luciferase gene construct, to evaluate Nrf2/ARE activation (HepG2-ARE). HepG2 cells are a suitable model in the present work because they have a Nrf2 pathway that responds in a normal manner to Nrf2 activators [83], and do not have reported mutations in Nrf2/KEAP1. The stably transfected HepG2-ARE Nrf2 reporter gene cell line was kindly provided by Steven Simmons [84]. The HepG2 and HepG2-ARE cells were cultured and maintained by standard methods using Opti-MEM medium with 4% fetal bovine serum (FBS) and geneticin/penicillin/streptomycin.

### 2.3. Nrf2 Reporter Gene Assays

Briefly, the HepG2-ARE cells were seeded (20,000 cells in 400 μL of Opti-MEM medium, 4% FBS, with geneticin/penicillin/streptomycin) in 24-well plates and incubated at 37 °C with 10% CO_2_. After 24 h the cells were treated with individual agents or combinations of agents. To measure synergy, cells were treated with extracts of rosemary, ashwagandha, and luteolin combinations or with the corresponding concentrations of each agent individually. In the PB125 synergy example depicted in the present work, this was visualized by treating the cells with 0–22 µg/mL of PB125 extract or with the corresponding amounts of rosemary extract (0–15 µg/mL), ashwagandha extract (0–5 µg/mL), and luteolin (0–2 µg/mL) alone, and comparing the PB125 combination with the sum of the signals observed from the three ingredients measured individually. After an additional 18 h of incubation, the cells were lysed in their wells with 100 μL of a lysing buffer that contains 3.5 mM sodium pyrophosphate to stabilize light output by luciferase. A 20 μL aliquot of cell lysate was removed to a clear glass test tube, placed in a BD Monolight 3010 luminometer (BD Biosciences, San Jose, CA, USA), background luminescence was measured, then 50 μL of 1 mM luciferin was injected into the tube and luciferase-dependent chemiluminescence was measured and reported as Relative Light Units (RLU).

### 2.4. Gene Expression Assays

#### 2.4.1. Cell Culture and RNA Isolation

HepG2 cells were cultured overnight in 24-well plates with control vs. 16 μg/mL PB125 (as an extract of 50 mg/mL in 100% ethanol) treatment. For gene expression comparisons, HepG2 cells were also cultured in the same method with 12 μg/mL PB123, 40 μg/mL Protandim, and 1.5 μg/mL DBM (concentrations which gave comparable levels of Nrf2 activation). Briefly, the cultured cells were washed two times with PBS in the wells, then treated with Trizol, then total RNA was isolated. Total RNA was further purified by using the Qiagen RNeasy clean-up columns (QIAGEN Inc., Valencia, CA, USA).

#### 2.4.2. Microarray Assays

The RNA concentration of each sample was determined based on the absorbance at 260 nm (A260) using a NanoDrop spectrophotometer (Thermo Fisher Scientific, Waltham, MA, USA). The integrity of total RNA samples was examined by Agilent TapeStation 2200 (Agilent, Santa Clara, CA, USA). Transcriptional analysis was done at the University of Colorado Denver Genomics and Microarray Core facility. Per standard protocol, 150 ng of starting total RNA was converted to cDNA with the GeneChip 3′ IVT PLUS Reagent Kit (Affymetrix/Thermo Fisher Scientific, Waltham, MA, USA), following the manufacturer’s protocol. After standard sample labeling, each sample was hybridized to the Affymetrix PrimeView human gene expression assay followed by examination with an Affymetrix GeneChip Scanner 3000 (Affymetrix/Thermo Fisher Scientific, Waltham, MA, USA). Three independent cell culture experiments and microarray assays were performed for PB125 treatment.

Briefly, each transcript and variants are represented by the use of 9–11 perfectly matched (PM) probes. The Affymetrix GeneChip software program (Affymetrix/Thermo Fisher Scientific, Waltham, MA, USA) determines the intensity of expression for all genes on the array and provides pair-wise comparison between chips. All of the data are subjected to cluster analysis by comparing the patterns of gene expression between all experimental groups using Partek Genomics Suite (Partek, St. Louis, MO, USA). Informative clusters are analyzed and the genes in those clusters identified. Informative clusters and genes of interest are also analyzed using Ingenuity Pathway Analysis (IPA) to explore known and potential new pathways and gene interaction networks.

#### 2.4.3. Quantitative Reverse Transcription-PCR Assays

Expression of the three Nrf2-dependent genes *HMOX1*, *GCLM* and *SLC7A11* were quantified by quantitative reverse transcription-PCR (qRT-PCR) using standard techniques. Briefly, the same total RNA used for microarray assay was used for qRT-PCR using a One-Step RT-PCR kit (Bio-Rad Laboratories, Hercules, CA, USA) with SYBR green in a Bio-Rad CFX96 Real-Time PCR System (Bio-Rad Laboratories, Hercules, CA, USA) according to the manufacturer’s instructions. The delta-delta Ct method was used to calculate the relative fold gene expression in the samples. The following primers in Table 1 were designed and used for quantitative real-time PCR of the human genes shown.

#### 2.4.4. RNA-seq Assays

##### RNA-seq Library Preparation

A total of 200–500 ng of total RNA was used to prepare the Illumina HiSeq libraries according to manufacturer’s instructions for the TruSeq RNA kit (Illumina, San Diego, CA, USA). This kit first isolates mRNA from total RNA using polyA selection, then fragments and primes the mRNA for creation of double-stranded cDNA fragments which are subsequently amplified, size selected, and purified for cluster generation.

##### Sequencing

The mRNA template libraries were then sequenced as single pass 50 bp reads on the Illumina HiSeq 4000 platform (Illumina, San Diego, CA, USA) at the University of Colorado’s Genomics and Microarray Core Facility (Aurora, CO, USA). We sequenced at a depth that provides ~40M single-read 50 bases reads per sample.

##### mRNA-seq Profiling

Derived sequences were analyzed by applying a custom computational pipeline consisting of the open-source GSNAP [85] Cufflinks [86], and R for sequence alignment and ascertainment of differential gene expression [87]. In short, reads generated were mapped to the human genome (GRCH38) by GSNAP [85], expression (FPKM) derived by Cufflinks [86], and differential expression analyzed with ANOVA in R. Genes significant at a false discovery rate (FDR) < 0.05 were submitted to pathway analysis using Ingenuity Pathway Analysis (Qiagen, Germantown, MD, USA) to identify pathways of interest that were modified by PB125.

### 2.5. Protein Assays

Heme oxygenase-1 (HMOX1) protein was measured on HepG2 cell lysates by HMOX1 sandwich enzyme-linked immunosorbent assay (ELISA) using the Human HMOX1 PicoKine ELISA Kit (Boster Biological Technology, Pleasanton, CA, USA), according to manufacturer’s instructions, based on an anti-human HMOX1 capture antibody adherent to 96-well plate, an anti-human HMOX1 biotinylated antibody as the second antibody, and an avidin-biotin-peroxidase complex to link the peroxidase enzyme to the HMOX1 antibody sandwich. The chromogenic 3,3′,5,5′-tetramethylbenzidine (TMB) substrate was used to measure peroxidase activity, and then the HMOX1 protein levels in the cell lysate samples were quantitated using a standard curve generated with human HMOX1 protein dilutions. Total protein in the lysates was measured using the method of Lowry [88].

### 2.6. Assays to Measure Cytoprotective Effects

Experiments were conducted to evaluate protective effects of PB125 pretreatment of HepG2 cells against cumene hydroxide-induced cytotoxicity, assessed by measuring changes in cell viability and changes in lactate dehydrogenase (LDH) release. 

#### 2.6.1. Cell Viability

Cell viability was measured using a cell counting kit-8 (CCK8) assay (Dojindo Molecular Technologies, Inc., Rockville, MD, USA) based on a water-soluble tetrazolium salt. Briefly, cells were seeded into 96-well plates at 10,000 cells/well and cultured overnight. After the indicated treatments, CCK8 solution was added to each well according to the manufacturer’s instructions and incubated for 2 h at 37 °C. Absorbance was measured at 450 nm using a microplate spectrophotometer (Bio-Tek, Winooski, VT, USA). Absorbance values were normalized to control-treated cells, and the data was reported as percentage viable cells relative to control-treated cells.

#### 2.6.2. LDH Release

LDH release was measured using an LDH assay kit assay (Cytotoxicity LDH Assay Kit-WST, Dojindo Molecular Technologies, Inc., Rockville, MD, USA). Briefly, cells were seeded into 96-well plates at 10,000 cells/well and cultured overnight. After the indicated treatments, LDH levels in solution were measured according to the manufacturer’s instructions. Absorbance was measured at 490 nm using a microplate spectrophotometer (Bio-Tek, Winooski, VT, USA). Absorbance values were compared to lysed cell values of LDH in the media, and were reported as % release relative to control-treated cells.

### 2.7. Statistical Analysis

The data are presented as mean ± standard error of the mean (SEM). Significant differences were determined by one-way ANOVA and post hoc Tukey multiple comparisons testing, or by Student’s *t* test for unpaired data using Prism software (GraphPad Software, San Diego, CA, USA). A *p* value < 0.05 was considered statistically significant.

## 3. Results

### 3.1. Synergy

**Figure 1:**
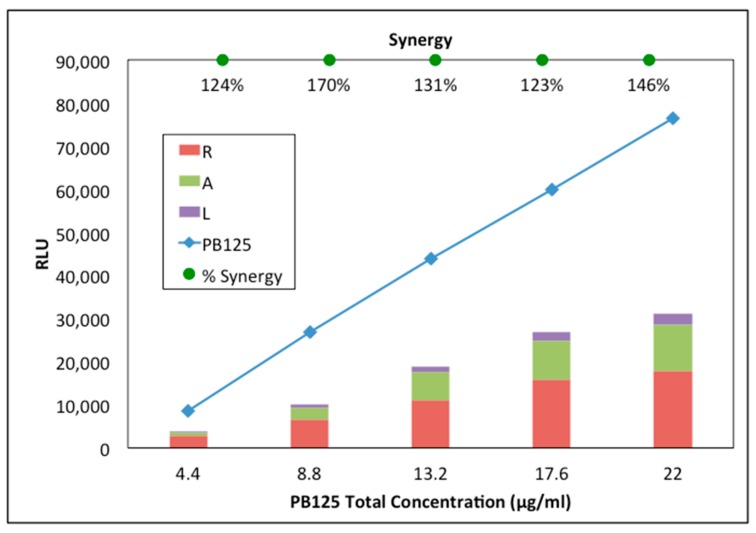
Synergistic activation of the Nrf2 pathway by the components in PB125. Synergy between the components of PB125 was observed measuring the relative light units (RLU) of chemiluminescence observed with added luciferin after ARE-driven luciferase gene expression was induced by treatment with range of concentrations of PB125 in the HepG2 (human liver) cancer cell line stably transfected with an ARE-driven luciferase gene as a promoter-reporter construct [84]. The individual contributions of the rosemary (R), ashwagandha (A), and luteolin (L) at the same concentration as in the 15:5:2 by mass combination of the same ingredients did not add (stacked bars) to as high of an RLU reading (Nrf2-dependent expression of the luciferase gene) as when cells were treated with the same amounts combined (*p* < 0.001).

Using the stably transfected ARE-promoter/luciferase-reporter HepG2 cell line [84], we determined that the rosemary, ashwagandha, and luteolin components of PB125 each exhibit Nrf2 activation, but, notably, that they work synergistically (much greater than the sum of their individual activities), to activate the Nrf2 transcription factor pathway. In our approach, we first determined that combinations of the rosemary and ashwagandha extracts gave highest Nrf2 activation at a 3:1 ratio, then combined that mixture with differing amounts of luteolin to and determined that 15:5:2 was optimal. To visualize the synergy of this 15:5:2 ratio (PB125), we treated HepG2-ARE cells with PB125 extract (0–22 µg/mL), rosemary extract (0–15 µg/mL), ashwagandha extract (0–5 µg/mL), or luteolin (0–2 µg/mL) alone, then compared the signal from the PB125 combination with the sum of the signals observed from the three ingredients measured individually (Figure 1).

### 3.2. Gene Expression

#### 3.2.1. HepG2 Gene Expression by Microarray

Affymetrix genechip microarray analysis revealed that canonical Nrf2 regulated genes *HMOX1*, *GCLM*, and *SLC7A11* were upregulated in HepG2 cells treated with PB125 (16 μg/mL, 24 h) compared to untreated controls (see Table 2), along with other canonical Nrf2 regulated genes including *GCLC*, *NQO1*, *TXNRD1*, *SQSTM1*, and *GSTA2*. All of these genes were also upregulated by the other two Nrf2 activators examined in the present study, PB123 and dibenzoylmethane (DBM), and with Protandim as previously described [82].

#### 3.2.2. HepG2 Gene Expression by qPCR

Follow-up experiments using quantitative PCR (qPCR) on 3 selected Nrf2-dependent genes (*HMOX1*, *GCLM*, and *SLC7A11*) in HepG2 cells treated with PB125 corroborated the upregulation observed using microarray (Table 2).

#### 3.2.3. HepG2 RNA-seq Gene Expression Quantitation

**Figure 2:**
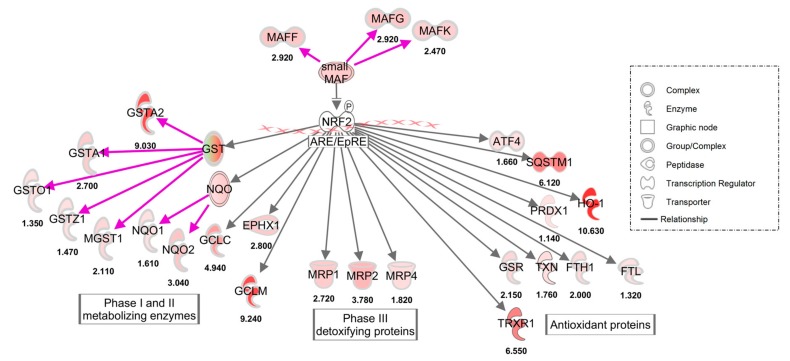
Ingenuity Pathway Analysis (IPA) indicates that PB125 activates the Nrf2 transcription factor pathway.

To further demonstrate and quantify gene expression changes caused by PB125, we utilized the RNA-seq approach to measure gene expression levels using separately cultured HepG2 cells. Evaluation of the dataset using Ingenuity Pathway Analysis (IPA) clearly demonstrated the primary importance of the Nrf2 transcription factor pathway in the gene expression changes caused by HepG2 cell treatment with PB125 (Figure 2).

The RNA-seq assays confirmed and quantified our findings that the Nrf2-dependent *HMOX1*, *GCLM*, and *SLC7A11* genes were significantly upregulated by PB125 treatment (*p* < 0.01). The data is shown in Table 2, showing the fold induction by PB125 compared to vehicle-treated control cells.

As shown in Table 2, the microarray, qPCR, and RNA-seq results were in agreement, with significant increases in *HMOX1*, *GCLM*, and *SLC7A11* gene expression, as characteristic Nrf2-dependent genes.

Nrf2 activation results in both up and down regulation of a variety of genes [89]. For example, in the present study the expression of 637 genes was increased and 686 genes was decreased by >2-fold in HepG2 cells treated overnight with 16 μg/mL PB125, determined by RNA-seq on triplicate samples. More is known about the direct effects of Nrf2 on increasing expression of cytoprotective genes, but Liu et al. very recently discovered a direct mechanism by which Nrf2 can form a complex with replication protein A1 and decrease gene expression; they identified a regulatory element adjacent to the 3′ end of the ARE in the myosin light-chain kinase (*MYLK*) gene that allows Nrf2 to function directly as a transcriptional repressor for the *MYLK* gene [90]. Related to this recent work, in our HepG2 RNA-seq dataset we found that PB125 treatment causes a statistically-significant decrease in *MYLK* gene expression (fold induction = −7.8, *p* < 0.0001), which fits with the repression effect described by Liu et al. [90].

#### 3.2.4. Non-Canonical Nrf2 genes and Non-Nrf2 Genes

As noted above, PB125 induced Nrf2 activation and upregulation of canonical Nrf2-dependent genes, and IPA identified the key involvement of the Nrf2 pathway for gene expression changes induced by PB125. Notably, in the microarray and RNA-seq experiments regulation was also observed, both up and down, of a large number of genes that appear to be Nrf2 regulated but that have not been previously reported to be under direct transcriptional control of Nrf2, as well as genes that may not be regulated by Nrf2 at all (Table 3). When dealing with plant extracts or even with pure single compounds, one cannot assume just because Nrf2 is activated by the extract or compound, that every gene modulated is therefore regulated by Nrf2. To shed more light on this issue we have compared the effects of multiple and diverse Nrf2 activators on each gene in question. If a gene is consistently up (or down) regulated by multiple known Nrf2 activators it seems most likely that the gene is indeed directly or indirectly modulated by Nrf2. If, however, very different effects are noted with multiple known Nrf2 activators (i.e., upregulation versus downregulation versus no effect) then the gene is most likely responding to a different transcription factor that happens to be activated by one or more, but not all members of the group of extracts and compounds examined. The group of genes with statistically significant expression changes induced by PB125 that appear likely to be dependent on Nrf2, but have not yet been reported to have expression changed by Nrf2 activators, includes genes of interest due to their potential health benefits when upregulated (such as *C9orf72*, *CCPG1*, *CYH*, *GCKR*, *LRP10* or *NCF2*) or downregulated (such as *DKK1*, *FABP1*, *FMO5*, *HMGCR*, *LEAP2* or *PCSK9*). In this group, it is worth noting that *FABP1* has been previously shown to be downregulated by genetic (Keap1 knockout) activation of Nrf2, but not chemical activation of Nrf2 [91]. The group of genes with statistically significant expression changes induced by PB125 that are likely due to other, non-Nrf2-related effects of PB125 also includes genes of interest due to their potential health benefits when upregulated (such as *VGF, MAT1A, PLAU, IL4R, CBS,* and *NOS3*) or downregulated (such as *APOL1, IFIT1, YPEL3.* and *CYP1A1*).

Table 3 shows a group of genes that we have seen to be consistently modulated by a four different Nrf2 activators that we have studied. These include PB125, PB123 (a similar composition containing rosemary, ginger, and luteolin) [81], Protandim^®^ (five component phytochemical composition [82], and dibenzoylmethane (DBM) [92]. As seen in Table 3, all four of these diverse Nrf2 activators are consistent in whether they upregulate or downregulate each of these 12 genes, which have not previously been suggested to be Nrf2 regulated.

In contrast, Table 4 shows a group of eleven genes that are not consistently modulated in HepG2 cells by four different Nrf2 activators. The numbers reflect fold-induction of the respective mRNA levels. In every case these genes were regulated in the opposite direction (or not regulated at all) by at least one of the four Nrf2 activators (upregulation shaded in red; downregulation shaded in green).

### 3.3. HMOX1 Protein ELISA

**Figure 3:**
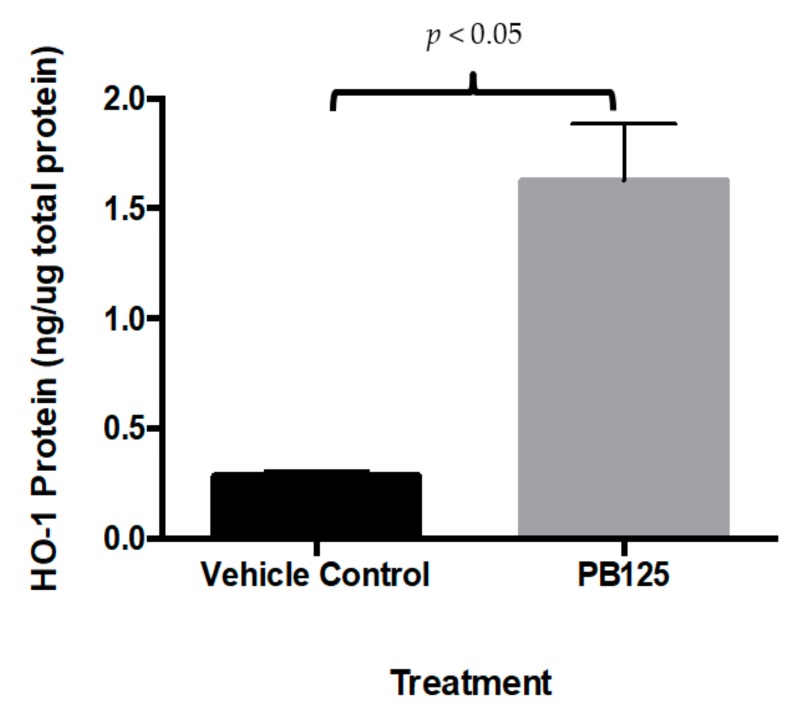
HMOX1 protein was increased by PB125. Treatment of HepG2 cells with PB125 increased the levels of HMOX1 protein determined by ELISA, as expected based on the large PB125-induced increase in *HMOX1* gene expression measured by microarray, quantitative PCR, and RNA-seq methods.

As anticipated by the increased *HMOX1* gene expression level following PB125 treatment, intracellular HMOX1 protein levels were concomitantly increased in HepG2 cells that were treated with overnight with 5 μg/mL PB125 (Figure 3).

### 3.4. Functional Benefit

To assess cell defense under challenge, we utilized the oxidant cumene hydroperoxide (CHP) to expose HepG2 cells to oxidative stress, with or without Nrf2 activation pretreatment with PB125. Overnight treatment with PB125 at 5 μg/mL was first shown to be nontoxic to HepG2 cells, then HepG2 cells were cultured with 5 μg/mL PB125 or vehicle control overnight, then the PB125 was removed and the cells washed with PBS prior to adding fresh culture media. Next the cells were challenged with cumene hydroperoxide for 6 h and cell injury assayed by measuring cell viability and LDH release.

**Figure 4:**
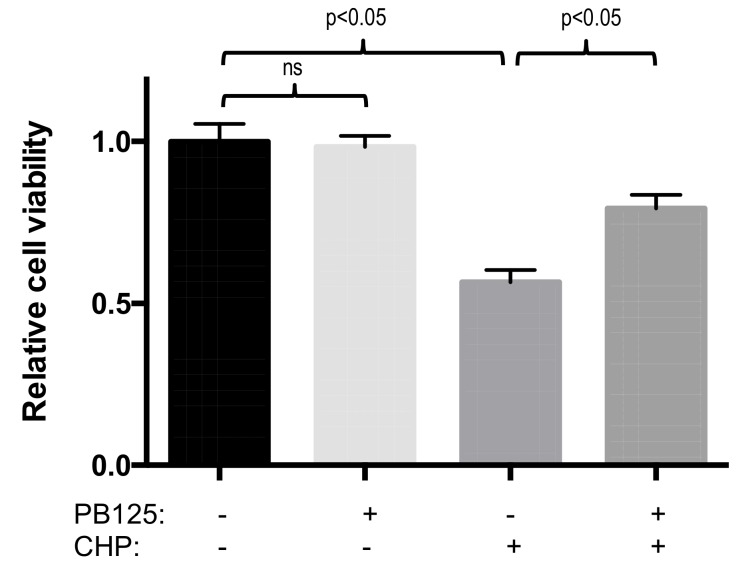
PB125 prevented oxidative stress-induced loss of cell viability. Cytotoxicity was not observed (cell proliferation measured by CCK8 assay) in HepG2 cells treated overnight with 5 μg/mL PB125 compared to vehicle control blank. In HepG2 cells cultured for 18h with 5 μg/mL PB125 or vehicle control, from which the culture media was removed and replaced and the cells were challenged with an oxidative stress by treatment with 25 μM cumene hydroperoxide (CHP) or untreated control for 6 h, cell toxicity (loss of viability) was caused by cumene hydroperoxide treatment but this toxicity was partially attenuated (*p* < 0.05) by PB125 pretreatment. ns: not significant.

**Figure 5:**
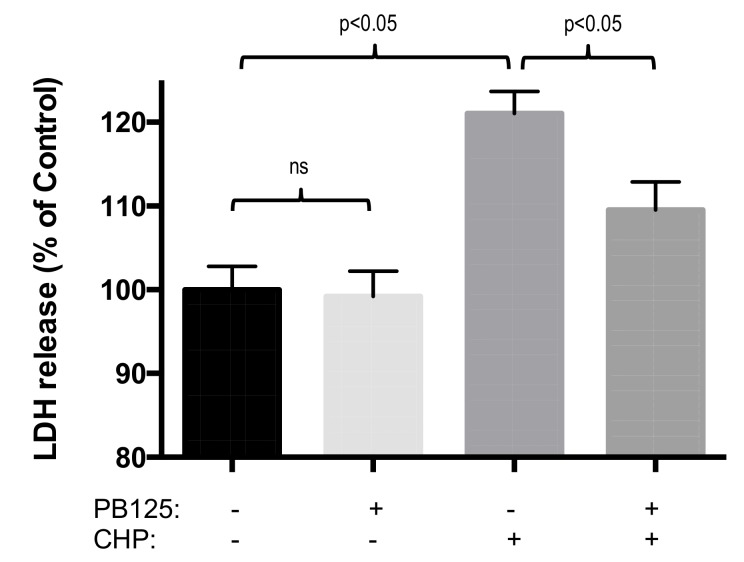
PB125 prevented oxidative stress-induced cell injury. Cellular injury (release of LDH into the culture media) was not observed in HepG2 cells treated overnight with 5 μg/mL PB125 compared to vehicle control blank. In HepG2 cells cultured for 18h with 5 μg/mL PB125 or vehicle control, from which the culture media was removed and replaced and the cells were challenged with an oxidative stress by treatment with 25 μM cumene hydroperoxide (CHP) or untreated control for 6 h, cell injury (release of LDH) was caused by cumene hydroperoxide treatment but this injury was partially attenuated (*p* < 0.05) by PB125 pretreatment.

Overnight treatment of HepG2 cells with 5 μg/mL PB125, a dose found to be relevant for gene expression, did not cause toxicity effects. The PB125 treatment was not cytotoxic as determined by cell viability measured by the CCK8 cell proliferation assay (Figure 4), and did not induce cellular injury measured by release of LDH into the culture media (Figure 5).

Pretreatment with 5 μg/mL PB125 protected against oxidative-stress-induced loss of viability in HepG2 cells that were subsequently challenged with 25 μM cumene hydroperoxide (Figure 4). In addition, pretreatment with 5 μg/mL PB125 decreased oxidative stress-induced cell damage (LDH release) caused by cumene hydroperoxide challenge of HepG2 cells (Figure 5).

## 4. Discussion

The ability to respond to stress-induced changes in gene expression has been observed to decline with aging in animals [93] and in humans [27]. Hepatic levels of Nrf2 decline in aged rats despite evidence of increased oxidative stress and inflammation, and the decline has been linked to an age-related and disease-related changes in expression of several microRNAs, including mir-146a [94,95], mir-34a and mir-93 [96], and mir-27a [97]. Carnosic acid/carnosol, the principal active ingredient in PB125, has specifically been shown to suppress expression of mir-34a, reducing oxidative stress and protecting against non-alcoholic fatty liver disease [98]. The age-dependent loss of Nrf2 provides a rational basis for restoring Nrf2-dependent gene expression in aging individuals by supplementing dietary intake of natural Nrf2 activators, thereby compensating for the loss of available Nrf2 by activating a greater fraction of what remains. We believe this to be a logical and safe approach to forestall the age-related changes in expression of Nrf2-regulated genes that cause or contribute to literally scores of well-studied diseases. The process of designing an appropriate Nrf2-activating supplement is complicated somewhat by the fact that, while numerous phytochemicals have been found capable of activating Nrf2 qualitatively in vitro, they differ considerably in many ways including in the concentrations that must be achieved for adequate modulation of gene expression. In addition, Nrf2-activating phytochemicals differ in the things they do that are unrelated to Nrf2, including changing the expression levels of non-Nrf2 genes (see Table 4).

In the present work, we found that the components of PB125 work together synergistically (Figure 1). Nrf2 activation pathways have been shown to be complex, involving not simply the release of Nrf2 from its cytosolic inhibitor Keap1 that targets it for degradation (known as the *canonical* pathway), but also additional mechanisms including phosphorylation of Nrf2 at Ser40 by protein kinase C and perhaps other kinases as an alternative means of freeing Nrf2 from Keap1 and/or increasing its half-life in the cell [99]. A more recent review of the many mechanisms that may contribute to Nrf2 activation includes potential roles for at least nine additional transcription factors, for epigenetic modifications involving methylation and acetylation of the Nrf2 gene, for post-transcriptional regulation by as many as 15 miRNAs, and for post-translational regulation via ubiquitination and proteasomal degradation by at least five proteins [100]. This elaborate network of control mechanisms not only underscores the evolutionary importance of the Nrf2 pathway, but also helps explain why combinations of activators that work at different control points in the network can often provide great synergy, far exceeding the effect of any single activator. For example, as seen in Figure 1, even though the molar concentration of withaferin A is much less than that of carnosic acid, its contribution to Nrf2 activation is quite substantial at about 60% that of carnosic acid, and after the effects of synergy, the activation is about 4.6 times that of carnosic acid alone. We believe that the advantage of a multi-component composition derives from combining ingredients that all activate Nrf2, but by different pathways.

Nrf2 regulates the gene expression of a wide variety of cytoprotective phase II detoxification enzymes and antioxidant enzymes through an enhancer sequence known as the antioxidant-responsive element (ARE) [15,16,17,18,19,20,21,22,23]. In the present work we found that PB125 potently induced Nrf2 activation, as did several other compositions used for comparison. This was observed using cells transfected with a Nrf2-driven promoter/reporter construct, by qRT-PCR, and also by global examination of genes as assessed by GeneChip and RNA-seq analyses. The canonical genes that are widely studied and accepted as Nrf2-regulated genes were consistently modulated by all three of the multi-component compositions and by DBM, as expected. Among the non-canonical genes that were consistently modulated by all the Nrf2 activators in the present work, some of those listed in Table 3 are considered by us to be worthy of additional discussion here because the Nrf2 transcription factor pathway has not previously been widely noted for its involvement with these genes.

*C9orf72* is now believed to be the largest single genetic contributor to amyotrophic lateral sclerosis (ALS), accounting for perhaps 40% of familial ALS and 10% of sporadic ALS. While the relationship to ALS is still a bit murky, Lall and Baloh [101] have reviewed several models, one of which is based on the 50% under expression of *C9orf72* observed in frontal cortex of ALS affected individuals, and the observations that *C9orf72* deletion or knockdown in two animal models leads to motor neuron degeneration. As PB125 upregulated *C9orf72* expression by 3.1-fold, this suggests that further study might be warranted.

The amyloid-beta (Aβ) peptide that accumulates in Alzheimer’s disease (AD) is derived from amyloid precursor protein (APP) following proteolysis by β- and γ-secretases. Low density lipoprotein receptor-related protein 10 (LRP10) is a functional APP receptor involved in APP trafficking and processing which interacts directly with APP to induce the accumulation of mature APP in the Golgi, reducing its presence at the cell surface and its processing into Aβ. Knockdown of LRP10 expression increases Aβ production, and expression of LRP10 is significantly lower in the post-mortem brain tissues of AD patients [102]. LRP10 was induced by PB125 2.5-fold by GeneChip and 3.7-fold by RNA-seq, suggesting a possible therapeutic role in AD.

Two genes involved in cholesterol metabolism are noteworthy. 3-Hydroxy-3-methylglutaryl- CoA reductase (HMGCR) catalyzes the rate-limiting step in the biosynthesis of cholesterol and is the target of the statin family of drugs. HMGCR was downregulated -2.8-fold by PB125. Cholesterol is carried in the plasma mainly by low-density lipoprotein (LDL), which is removed by the LDL receptor (LDLR) in the liver for excretion. The LDLR may then be recycled. Proprotein convertase subtilisin/kexin type 9 (PCSK9), however, can bind to the LDLR, causing it to be degraded rather than recycled, with the effect of slowing cholesterol removal. Blocking this action of PCSK9, increases cholesterol removal. When cholesterol synthesis is inhibited (as by a statin) the body compensates by increasing PCSK9 synthesis to conserve cholesterol [103], possibly contributing to the statin-resistance seen in some people. Hence, we have the appearance of a new class of drugs—the PCSK9 inhibitors. Thus, a statin combined with a PCSK9 inhibitor will slow the synthesis and increase removal of cholesterol. PB125, similarly, would appear to slow cholesterol synthesis by downregulating HMGCR (−2.8-fold) while also increasing clearance by downregulating PCSK9 (−4.4-fold). Once again, these results suggest a possible role for Nrf2 activators in the regulation of cholesterol levels, but have not been clinically investigated.

The group of genes in Table 4 are not thought to be directly regulated by Nrf2 owing to the lack of consistent regulation in the same direction by the four known Nrf2 activators examined, and are thus assumed to be regulated by other factors which may be unique to one or more of the activators. Some of these genes are also noteworthy.

*APOL1* encodes apolipoprotein L1, a component of LDL. *APOL1* has substantial clinical significance for people of recent African ancestry because of two common variants that covey resistance to infection by *Trypanosoma brucei rhodesiense*, but which later lead to kidney failure [104]. Due to the widespread occurrence of these toxic gain-of-function mutations, African Americans have end stage kidney failure at rates five times that of European Americans. Olabisi et al. have proposed that nephropathy may be mediated by *APOL1* variant-induced loss of intracellular K^+^ and aberrant activation of stress-activated protein kinase signaling [105]. Affymetrix GeneChip analysis showed *APOL1* was downregulated by PB125 and PB123, but not by the other two Nrf2 activators (see Table 4). RNA-seq analysis showed that PB125 downregulated *APOL1* by 2.4 fold. The possibility of a clinical therapeutic effect via downregulation of the mutant toxic forms of *APOL1* merits further investigation.

Betaine-homocysteine *S*-methyltransferase (BHMT), cystathionine beta-synthase (CBS), and methionine adenosyltransferase 1, alpha (MAT1A) are noteworthy because all are involved in methionine/homocysteine metabolism. MAT1A is essential for the formation of *S*-adenosylmethionine, the methyl donor required by numerous metabolic pathways throughout the body. BHMT is one of two enzymes capable of re-methylating homocysteine back to methionine to continue the cycle. CBS is essential for catabolizing the excess homocysteine produced by the cycle. CBS is view as particularly important as its deficiency is the most common cause of hyperhomocysteinemia [106]. CBS is important not only for its role in the detoxification of homocysteine, but also for its production of hydrogen sulfide, which is itself a Nrf2 activator and neuroprotective agent [107]. All three of these enzymes are upregulated by PB125 (Table 4).

CYP1A1 is a phase I cytochrome *P450* gene involved in metabolism of xenobiotics and drugs, and its expression is largely controlled by the aryl hydrocarbon receptor (AhR), a transcription factor activated by binding of organic pollutants, including polycyclic aromatic hydrocarbons and dioxins. There has been a longstanding perception that CYP1A1 inducers are automatically disqualified from pharmaceutical development due to the ability of CYP1A1 to convert benzo[a]pyrene, a fairly common environmental pollutant found in charred meat and cigarette smoke, into potent human carcinogens [108]. Other studies, however, have provided a more complex picture of the roles of CYP1A1, with the observation of cytoprotective effects which depend upon particulars such as organs targeted and mode of administration of benzo[a]pyrene [109,110]. The effects of CYP1A1 are not confined to detoxification versus activation of carcinogens. It also appears to promote smoking-induced bone loss, or osteoporosis [111]. A common symptom of dioxin toxicity is an acne-like condition called chloracne. It was recently found that agents such as cinnamaldehyde that both inhibit CYP1A1 expression and activate Nrf2 are efficacious treatments for chloracne [112]. These properties are shared by both PB125 and PB123 (Table 4).

*VGF* encodes a 68 kDa polypeptide, comprising 615 amino acids, that is cleaved to ten or more polypeptides which have been associated with a number of neuroendocrine roles [113]. While the exact functions of the peptides are in most cases still undetermined, it has been noted that in cerebrospinal fluid from ALS patients, immunoreactivity of the full-length *VGF* was reduced in parallel with development of ALS symptoms, leading the authors to hypothesize that restoring VGF expression in spinal cord motor neurons could therapeutically benefit clinical ALS [114]. VGF serum levels are significantly lower in patients with major depressive disorder compared to controls and were reversed by 8 weeks of drug treatment with anti-depressants [115]. Exercise has been argued to enhance cognitive function and slow progressive neurodegenerative disease. Alvarez-Saavedra et al. have studied a mouse with a conditional knockout of the *Snf2h* gene which impairs cerebellar development, producing mice with poor motor function, progressive ataxia, and death between postnatal days 25 and 45 [116]. They showed that voluntary running induced an endogenous brain repair mechanism that resulted in an increase in hindbrain myelination and in long-term survival of *Snf2h*-cKO mice. VGF production was a major cause of this effect. *Snf2h*-cKO mice treated with full-length *VGF*-encoding adenoviruses removed the requirement of exercise for survival. These results suggest that increased expression of *VGF* might represent a therapeutic strategy for cerebellar ataxia and other pathologies of the central nervous system. [116]. Also of interest is the pleiotropic nature of the *VGF* gene. Stephens et al. found that VGF regulates secretory granule formation in pancreatic islet beta cells. *VGF* loss-of-function studies in both isolated islets and conditional knockout mice caused a profound decrease in stimulus-coupled insulin secretion. Moreover, *VGF* was required for exit of granule cargo from the trans-Golgi network and for proinsulin processing. It also functioned to replenish insulin granule stores following nutrient stimulation. The studies suggest that loss of *VGF* could be involved in the development of islet beta cell dysfunction in type 2 diabetes [117]. Our gene expression data show that three of the four compositions we examined induced VGF, with PB125 showing a five-fold induction by RNA-seq (Table 4).

## 5. Conclusions

We have constructed a potent Nrf2 activating composition that combines several novel features in addition to the synergistic interactions we have previously described [38]. A key aspect in the design of PB125 involved selection of phytochemical ingredients based on the recognition that the Nrf2 pathway consists not only of an “activation” pathway that provides for nuclear translocation of Nrf2, but also of a second “deactivation” or “shutdown” pathway to bring the process to an appropriate end. We believe that PB125′s Nrf2-activating properties can assist in restoration of the Nrf2 activation that otherwise can diminish with aging. We found that pretreatment with PB125 protected cultured HepG2 cells against an oxidative stress challenge caused by exposure to the organic peroxide cumene hydroperoxide. The Nrf2 activation, gene expression changes, and protection against oxidative stress by the dietary supplement PB125 support its use to promote healthy aging.

## Figures and Tables

**Table 1:** Primer pairs for PCR of characteristic Nrf2-dependent genes and GAPDH housekeeping gene.

Primer	Sequence
*GAPDH*, forward	5′ GGACCTGACCTGCCGTCTAG 3′
*GAPDH*, reverse	5′ GAGGAGTGGGTGTCGCTGTT 3′
*HMOX1*, forward	5′ GACAGCATGCCCCAGGATT 3′
*HMOX1*, reverse	5′ GTGGTACAGGGAGGCCATCA 3′
*GCLM*, forward	5′ TTGCCTCCTGCTGTGTGATG 3′
*GCLM*, reverse	5′ GTGCGCTTGAATGTCAGGAA 3′
*SLC7A11*, forward	5′ TGGGCTGATTTATCTTCGATACAA 3′
*SLC7A11*, reverse	5′ ATGACGAAGCCAATCCCTGTAC 3′

**Table 2:** Upregulation of selected canonical Nrf2 regulated genes and verification of gene expression upregulation of those genes by microarray, quantitative PCR (qPCR) and RNA-seq assays in HepG2 cells treated with PB125 (qPCR data normalized to GAPDH, average from two samples for each group)(RNA-seq data mean ± SEM, *n* = 3 for PB125 and untreated groups, *p* < 0.01 for each gene). Treatment of cultured HepG2 cells with PB125 (16 μg/mL) increased gene expression of *HMOX1*, *GCLM*, and *SLC7A11*.

Gene	Gene Name	Fold Change by Microarray	Fold Change by qPCR	Fold Change by RNA-seq
*HMOX1*	heme oxygenase (decycling) 1	2.6	2.6	10.6 ± 0.3
*GCLM*	glutamate-cysteine ligase, catalytic subunit	5.4	8.5	9.2 ± 0.1
*SLC7A11*	solute carrier family 7 (anionic amino acid transporter light chain, xc- system), member 11	4.4	8.6	9.5 ± 0.3

**Table 3:** Non-canonical Nrf2 genes that are consistently modulated in HepG2 cells by four different Nrf2 activators. The numbers reflect fold-induction of the respective mRNA levels based on averages of 3 independent microarray or RNA-seq experiments for PB125 treatment (16 μg/mL), and single microarray experiments for the comparison treatments (PB123, Protandim, and dibenzoylmethane (DBM)). In every case these twelve genes were regulated in the same direction by all four Nrf2 activators (upregulation shaded in red; downregulation shaded in green).

Gene	Gene Description	Nrf2 Activators			
		PB125	PB123	Protandim	DBM
*C9orf72*	C9orf72	3.1	3.9	1.8	1.2
*CCPG1*	Cell cycle progression 1	5.2	3.6	4.8	2.3
*CTH*	Cystathionine gamma-lyase	7.2	8.0	4.5	1.4
*GCKR*	Glucokinase (hexokinase 4) regulator	4.0	23.6	1.8	2.2
*LRP10*	Low density lipoprotein receptor-related protein 10	2.5	5.1	2.4	1.3
*NCF2*	Neutrophil cytosolic factor 2	2.4	1.5	1.5	1.3
*DKK1*	Dickkopf WNT signaling pathway inhibitor 1	−9.5	−21.4	−2.1	−1.4
*FABP1*	fatty acid binding protein 1, liver	−6.5	−16.7	−7.7	−2.7
*FMO5*	Flavin containing monooxygenase 5	−3.3	−14.3	−3.2	−1.5
*HMGCR*	3-Hydroxy-3-methylglutaryl-CoA reductase	−2.8	−6.3	−2.0	−1.5
*LEAP2*	Liver expressed antimicrobial peptide 2	−5.8	−1.75	−4.8	−2.3
*PCSK9*	Proprotein convertase subtilisin/kexin type 9	−4.4	−1.6	−5.3	−1.6

**Table 4:** Genes that are inconsistently regulated by four different Nrf2 activators. The numbers reflect fold-induction of the respective mRNA levels based on averages of 3 independent microarray or RNA-seq experiments for PB125 treatment (16 μg/mL), and single microarray experiments for the comparison treatments (PB123, Protandim, and DBM). In no case was one of these eleven genes regulated in the same direction by all four Nrf2 activators (upregulation shaded in red; no effect in yellow; downregulation shaded in green).

Gene	Gene Description	Nrf2 Activators			
		PB125	PB123	Protandim	DBM
*APOL1*	Apolipoprotein L1	−1.4	−1.3	1.5	1.3
*BHMT*	Betaine-homocysteine S-methyltransferase	2.5	6.9	1.0	1.4
*CBS*	Cystathione beta-synthase	1.4	2.4	−2.1	1.0
*CYP1A1*	Cytochrome P450 family 1 subfamily A member 1	−1.4	−1.6	9.2	6.7
*IFIT1*	Interferon induced protein with tetratricopeptide repeats 1	−2.1	−1.4	3.1	−1.3
*MAT1A*	Methionine adenosyltransferase I, alpha	1.3	2.3	−1.8	1.7
*NOS3*	Nitric oxide synthase 3	1.7	1.2	1.0	1.6
*PLAU*	Plasminogen activator, urokinase	2.9	1.7	−3.8	−1.1
*TP53INP1*	Tumor protein p53 inducible nuclear protein 1	−1.9	−5.3	1.2	−1.3
*VGF*	VGF nerve growth factor inducible	5.0	2.0	1.0	1.2
*YPEL3*	Yippee like 3	−1.4	−2.0	1.2	−1.2
